# Personality traits and self-control: The moderating role of neuroticism

**DOI:** 10.1371/journal.pone.0307871

**Published:** 2024-08-21

**Authors:** Fredrik A. Nilsen, Henning Bang, Espen Røysamb

**Affiliations:** 1 Department of Psychology, University of Oslo, Oslo, Norway; 2 Department of Leadership of Land Operations, The Norwegian Defense University College, Oslo, Norway; 3 Department of Psychology, PROMENTA Research Center, University of Oslo, Oslo, Norway; 4 Norwegian Institute of Public Health, Oslo, Norway; Imperial College London, UNITED KINGDOM OF GREAT BRITAIN AND NORTHERN IRELAND

## Abstract

Self-control is important for mental and physical health, and personality traits are vital antecedents for self-control. Previous studies suggest that conscientiousness and extraversion enhance self-control, whereas neuroticism hampers it. However, the link between personality and self-control has mostly been studied using a narrow conceptualization of self-control, as the ability to resist impulses, thus excluding initiatory self-control. Also, no studies have examined whether and how personality traits interact with one another to increase, or reduce, self-control. Data were collected on two occasions from 480 military cadets (31.04% female) to examine the relationship between the Big Five personality traits and self-control (general, inhibitory, and initiatory self-control). Furthermore, the study investigated the moderating role of neuroticism, as a trait and as individual facets, on the relationship between the other personality traits and self-control. Although neuroticism correlated negatively with all self-control dimensions, there were unique relations only with general and inhibitory self-control. Extraversion correlated positively with all self-control dimensions but was only uniquely related to initiatory self-control. Conscientiousness correlated positively with all self-control dimensions and this pattern persisted when we assessed the unique effects. Openness to experience and agreeableness correlated positively with general and inhibitory self-control but had no unique effects on any of the self-control dimensions. Neuroticism negatively moderated the relationship between extraversion and both general and inhibitory self-control, and the relationship between conscientiousness and both general and initiatory self-control. The facet-level analysis confirmed the general patterns and provided further detail on which facets of neuroticism were the most influential as moderators. In conclusion, the study highlights the critical role of different types of self-control, and that neuroticism plays a cardinal role for the effects of conscientiousness and extraversion on self-control.

## Introduction

Trait self-control influences key facets of human functioning and has been linked to core aspects of life, such as health, life satisfaction, relationships, and work performance [1–8]. Identifying antecedents for self-control is therefore important to better understand and support the development of self-control [9]. Research on trait self-control has increasingly moved away from viewing self-control as a unidimensional concept, to seeing it as a multidimensional trait that includes both reactive and proactive processes [10, 11]. Traditionally, self-control has been considered a reactive process, employing effortful, *inhibitory self-control* (the intentional resisting of unwanted thoughts, feelings, and behavior) in the face of temptations that lead to dilemmas between smaller-immediate versus larger-delayed rewards [2]. However, recent trait self-control research has suggested an expansion to the traditional perspective by including a second and more proactive type of self-control: *initiatory self-control* [12–14]. This progress of the self-control concept has been supported by considerable evidence [13, 15]. Initiatory self-control refers to the proactive processes and strategies that individuals use to reach long-term goals when they anticipate temptations in conflict with these goals [10, 16]. These strategies allow individuals to be more agentic in their efforts, hence also conserving one’s inhibitory efforts, and are therefore considered “effortless” [17]. When such strategies are used at an early stage in the self-control process, the successful implementation of initiatory self-control may render inhibition unnecessary, thereby increasing self-control success by avoiding self-control effort [11] and/or depletion [18].

Duckworth and Seligman [19] addressed the need for “connecting the burgeoning literature on self-control to the equally fertile research on Big Five personality” (p. 716). A review of the literature shows that the relationship between personality traits and self-control has almost exclusively been studied from a perspective that considers self-control an inhibitory mechanism. A reason for this insufficiency may have been a lack of methods for assessing self-control as a multidimensional concept. However, recent advances in self-control assessment that separate inhibitory and initiatory self-control mechanisms as second-order factors [15, 16] permit the relationship between self-control and personality to be further clarified and potentially re-evaluated.

Previous studies on the relationship between self-control and personality have mostly studied this relationship by assessing personality variables in isolation from each other [2, 20]. Although personality traits are supposed to be independent, there is considerable support for the notion that traits partly overlap [21, 22]. Additionally, studies indicate that personality traits may interact. That is, the effect of a certain trait on an outcome may be dependent on the level of another trait. It has therefore been argued that studies of personality should consider the dynamics from the interactions of traits to avoid masking of multiplicative relationships between traits [23–25].

### The relationship between individual personality traits and self-control

The Five-Factor Model of personality is a widely accepted framework that categorizes human personality traits into five broad dimensions: neuroticism, extraversion, openness to experience, agreeableness, and conscientiousness [26, 27]. These traits capture key aspects of an individual’s behavioral tendencies, emotional responses, and interpersonal interactions, providing a comprehensive understanding of personality that can predict relevant criteria [28].

Identifying the individual relationships between the Five-Factor model’s personality traits and the types of self-control can be the first step in understanding how potential interactions between personality traits may affect self-control. The literature shows several studies on the relationship between personality traits and inhibitory self-control [i.e., 2, 20, 29–32], but only two studies in the literature examine the relationship between personality traits and initiatory self-control [16, 33]. Thus, we suggest that it is crucial to further explore this relationship to obtain a more precise understanding of the antecedents for self-control.

Studies show that conscientiousness and neuroticism are reported to be moderately to strongly associated with both inhibitory and initiatory self-control [2, 16, 33], while extraversion is found to be moderately to weakly related to inhibitory and initiatory self-control [2, 16, 20]. Agreeableness and openness to experience are either weakly or unrelated to both inhibitory and initiatory self-control [2, 16, 33]. In support, Tangney et al. [2] found that the relationship between agreeableness and inhibitory self-control became insignificant when controlling for social desirability. Identifying neuroticism, conscientiousness, and extraversion as the most significant personality traits for a person’s ability to exhibit self-control is consistent with the extant, broader literature on self-regulation [9, 34, 35].

Given the nature of neuroticism, extraversion, and conscientiousness, it is understandable that they often are singled out as the traits with the strongest relationships to self-control. Individuals high in neuroticism tend to experience more negative emotions, find it difficult to regulate emotions, and have heightened stress reactivity [36]. It is argued that this can lead to impulsive behaviors and a lack of self-control in stressful or emotionally charged situations [37]. Extraversion is characterized by, among other facets, positive emotions and assertiveness. People with high self-control are noted for being better equipped to amplify their positive emotions [38]. Furthermore, assertiveness may be a means to set and work toward long-term goals as it allows individuals to communicate their needs, opinions, and boundaries effectively [39]. Finally, conscientiousness and self-control are related as both focus on goal-directed behavior and responsible decision-making, but studies have also documented that self-control have discriminant and incremental validity beyond conscientiousness [2, 16, 20]. For instance, conscientiousness and self-control differ in terms of scope (i.e., conscientiousness includes traits such as organization, thoroughness, punctuality, and reliability) and specificity (self-control concerns regulating one’s reactions and behaviors in specific situations and prioritizing long-term benefits over immediate gratification).

### Neuroticism as a moderator for the relationship between other personality traits and self-control

To the best of our knowledge, no studies have examined how interaction between personality traits influence the personality traits’ relationship to self-control. However, theory suggest that neuroticism plays a fundamental role for self-control. The urgency-premeditation-perseverance-sensation seeking (UPPS) model [40] and the Carver model of impulse and constraints [41] are both considered to be two major personality models for self-control [11, 42], but the models differ in the regulatory role given to neuroticism [43]. The Carver model suggests that inhibition is driven by a *fear-based breaking mechanism* (neuroticism) that stops individuals from being impulsive. In contrast, the UPPS model suggests that negative affectivity (neuroticism) is encouraging impulsive behavior. In both these models, combining neuroticism with another trait is hypothesized to alter the influence of other personality traits. An explanation for the dominant role given to neuroticism in these self-control models may be based on the observation that neuroticism is positively related with increasing sensitivity of the fight-flight-freeze system and behavioral inhibition system [44]. These heightened levels of sensitivity are characterized by a greater perception of threats in one’s surroundings and a heightened fear of punishments, leading to negative thoughts, avoiding further unpleasant experiences, and ultimately, reducing subsequent goal-oriented behaviors [43, 45–47].

Research examining the interplay of personality traits on issues associated with self-control suggest specific prevailing trait interactions. In exploring various trait interactions, Vollrath and Torgersen [35] found that high neuroticism and low conscientiousness were the most likely trait combination for smokers. This result has been replicated by Terracciano and Costa [48] who showed that the high neuroticism and low conscientiousness trait interaction is three times more likely in smokers compared to non-smokers. The link between achievements and self-control is firmly established, and a meta-analysis showed that performance in the school/work domain is the domain where self-control behaviors show the largest effect sizes [49]. Conscientiousness is considered as the main trait for predicting job performance, while neuroticism and extraversion either play a small and insignificant role [50]. Nevertheless, an investigation into the interplay of personality traits and job performance revealed that the interaction of neuroticism and extraversion yielded effect sizes on par with conscientiousness [51].

### The present study

In this study, we first examined the relationship between personality traits and self-control by looking at bivariate correlations. Secondly, we examined the unique effect of individual personality traits on self-control by controlling for the remaining personality traits. Thirdly, we investigated to what extent levels of neuroticism, as a factor and as individual facets, moderated the relationship between other personality traits and self-control. For our first two aims, based on former studies of the relationship between personality traits and self-control [2, 16, 20, 33], we predicted that conscientiousness would have a positive, moderate relationship with self-control. Neuroticism was expected to show a moderately negative relationship with self-control, while extraversion was expected to relate positively, but moderately to weakly to self-control. Agreeableness and openness to experience were expected to be either weakly or unrelated with self-control. For our third aim, given the fundamental role of neuroticism [52, 53], its major negative affectivity-component [54], and the important role that negative affectivity appear to play as a regulator for self-control [40, 41, 55], we explored the extent to which varying levels of neuroticism differentially moderated the relationship between the other personality traits on the one hand and the dimensions of self-control on the other. Additionally, we delved into the individual facets of neuroticism to determine their impact on any of the interaction effects at the trait level. Since the literature is ambiguous about the regulatory role of neuroticism [41, 56], and its facets, this part of the study was exploratory.

## Methods

### Participants

Participants were army cadets from two cohorts (*n* = 480, 331 male and 149 female, *M*_age_ = 21.48 years, *SD*_age_ = 2.13) that were enrolled in a Bachelor of Arts program in military studies from 2018 to 2022. Inclusion criteria included giving consent to participate and being enrolled in the Bachelor of Arts program, while those that only participated in the first of the two surveys were excluded. Out of the 572 invitations, 504 participated in the first survey and 480 participated in the second survey. The participation rate for the first cohort (*n* = 504, 344 male and 160 female, *M*_age_ = 21.88 years, *SD*_age_ = 2.03) was 80.14%, and the participation rate for the second cohort was 89.94% (*n* = 480, 331 male and 149 female, *M*_age_ = 21.63 years, *SD*_age_ = 2.51), giving an overall participation rate of 84.09%. All participants had completed secondary education, had been found fit for conscription in Norway, and were screened by the same criteria for health and mental aptitude prior to admission to higher education in the Norwegian Armed Forces.

An a priori power analysis estimated that a minimum sample size of *n* = 395 was required for detecting “small effects” in the regression analyses (*f*^*2*^ = .02, *α* = .05, 1 - *β* = .80). Post-hoc analyses revealed that the study was powered at 0.87 to detect “small effects” (*f*^*2*^ = .02, *α* = .05). Power analyses suggested that the sample was sufficiently large to identify meaningful moderated relationships [57].

### Instruments

#### The NEO-personality assessment (NEO-PI-3)

The Norwegian version of NEO-PI-3 [39, 58] was used to measure personality traits. The 240 items of the scale measure 30 facets, which in turn are classified as five general personality traits: neuroticism, extraversion, openness to experience, agreeableness, and conscientiousness (see [59] for further information on factor structure). Participants responded to the items by indicating their agreement with the items on a five-point Likert scale (e.g., “I seldom feel nervous”) ranging from 1 (*strongly disagree*) to 5 (*strongly agree*). The estimated reliabilities for the personality factors were from good to excellent: neuroticism, *α* = .91, ω = .91; extraversion, *α* = .88, ω = .91; openness to experience, *α* = .90, ω = .92; agreeableness, *α* = .89, ω = .90; conscientiousness, *α* = .88, ω = .95. The estimated reliabilities for the neuroticism facets were acceptable: anxiety, *α* = .61, ω = .70; anger hostility, *α* = .71, ω = .78; depression, *α* = .65, ω = .64; self-consciousness, *α* = .66, ω = .68; impulsiveness, *α* = .68, ω = .70; vulnerability, *α* = .65, ω = .70.

#### The multidimensional self-control scale (MSCS)

The full, 29-item version of the MSCS [16] was used to measure self-control (e.g., “When it is hard to get started on a task, I try to find something to get me going”). Ten items of the scale are reversed-keyed. The MSCS assessed self-control with six first-order factors that make up two second-order factors; inhibitory self-control (procrastination, attentional control, and impulse control) and initiatory self-control (emotional control, goal orientation, and self-control strategies). Finally, the second-order factors aggregate to a third-order factor, general self-control. Participants reported how well each item described themselves using a scale ranging from 1 (*not at all*) to 5 (*very much*). The estimated reliabilities for the test scores of the variables applied in this study were from acceptable to good: general self-control, *α* = .87, ω = .83; inhibitory self-control, *α* = .86, ω = .83; initiatory self-control, *α* = .78, ω = .78.

#### Procedure

To mitigate common source bias, the study was conducted in two phases with a nine-week interval between them. The aim was to decrease participants’ inclination to use earlier responses as a reference for subsequent answers. The data for personality traits were obtained during the initial phase (time 1) and the data for self-control were collected nine weeks later (time 2).

Potential participants were informed about the voluntary nature of their participation, their rights as participants, and what participation would entail. The informants agreed to participate by signing a written informed consent form that was in line with the guidance of the Norwegian Agency for Shared Services in Education and Research. The study underwent a review and obtained approval from the Research Review Committee at The Norwegian Defense University College (Review NO. 2017919).

### Data analysis

Analyses for statistical power were conducted using G*Power 3.1.9.7 [60], while all other analyses were conducted by using R version 4.3.1 [61] and the packages *psych*, version 2.3.9 [62]; *ggplot2*, version 3.4.3 [63]; *car*, version 3.1.2 [64]; *lavaan*, version 0.6–18 [65] and *patchwork*, version 1.1.3 [66].

Given that the MSCS [16] is a fairly new instrument, we evaluated the latent structure. We employed a confirmatory factor analysis (CFA) with the maximum likelihood estimator with robust standard errors (MLR) as the estimation method. This choice was based on recommendations that suggest MLR should be used when ordinal data consists of five or more categories and can be treated as categorical data [67, 68]. Moreover, when the sample size reaches 500, the MLR-estimator outperforms the alternative weighted least squares mean and variance-estimator (WLSMV) by exhibiting lower levels of bias in standard errors of interfactor correlations. [69]. The model’s fit was assessed using four fit-indices: comparative fit index (CFI), Tucker-Lewis index (TLI), root mean square error of approximation (RMSEA), and standard root-mean-square residual (SRMR). According to commonly used criteria, acceptable fit is achieved when CFI is greater than 0.90, TLI is greater than 0.90, RMSEA is less than 0.08, and SRMR is less than 0.08. On the other hand, excellent fit is indicated by CFI greater than 0.95, TLI greater than 0.95, RMSEA less than 0.05, and SRMR less than 0.05 [70].

When using the term *effect* in the following, we refer to *statistical effects* and not necessarily causal effects. For the first aim, the relationship between personality traits and self-control were examined using Pearson’s *r*. For the second aim, the unique effect of individual personality traits on self-control, when controlling for the remaining personality traits, were assessed using multiple linear regression. For the third aim, interactions between personality traits and their relationship with self-control were assessed by a between-participant design using a moderated linear regression analysis [71]. This technique allowed us to explore how one variable, in this case, neuroticism, influenced the strength and direction of the relationship between other personality traits and self-control. Scores for personality and self-control were standardized using z-scores prior to computing the interaction term [72]. The moderator variable, neuroticism, was divided into terciles, representing the categories: “low”, “medium”, and “high”. This approach allowed us to examine non-linear effects and the potential interaction effects between unique levels of neuroticism with other traits on self-control. A potential limitation with categorizing continuous variables is a loss of information. However, when comparing a strategy that uses the upper and the lower terciles to a strategy that uses dichotomizing, the efficiency loss is reduced by 50% and “loses little in efficiency compared to linear regression on the original continuous predictor” [73].

For our moderated regression analyses, we first fitted a multiple linear regression model where a continuous focal predictor was assessed for its dependence on a categorical moderating variable using the category “low” as a reference level, for the moderation effect on the outcome variable. Some prior findings suggest that sex and age covaries with self-control [74] and the link between personality and self-control is well-established [2]. Hence, all interaction models were controlled for age, sex, and the remaining personality traits that currently were not included in the interaction term. For instance, openness to experience, agreeableness, and conscientiousness were introduced as control variables when we assessed how neuroticism moderated the relationship between extraversion and the different dimensions of self-control. Using two-way ANOVA, analyzing the reduction in the residual sum of squares, models with and without interaction terms were compared to determine if the nested model with interaction terms were significantly better than the base model at capturing the data. Once we determined the connections between personality traits and self-control dimensions that were influenced by neuroticism, we conducted an analysis to pinpoint which of the six facets of neuroticism had the greatest impact on these moderation effects.

### Controlling for common method bias and conceptual overlap between self-control and conscientiousness

Common method bias can be a concern when analyzing responses that have used the same assessment method. A single-factor test was used as a statistical remedy to control for the occurrence of common method bias [75, 76] between the data collected on the two different occasions. We added an unrotated first-order methods factor that includes the items of interest [77]. To set a strict criterion, we selected the 29 self-control items and the 30 conscientiousness items for this test. Results showed a shared variance of 17.23% for the first factor, thus being well below the 50% cut-off used to suggest influence from common method bias [77, 78].

We proceeded to examine the conceptual overlap between self-control and conscientiousness as a control for the jangle problem, i.e. different terms are used to refer to the same underlying construct [79]. By correlating self-control items with conscientiousness items, we discovered inter-item correlations ranging from *r* = -.13 to .31. When converted to absolute values, the average correlation is found to be *r* = .08 (*SD* = .06). Notably, the correlations between self-control and conscientiousness, at all levels, underscore the distinctiveness of self-control from conscientiousness (see also S1 Table for facet-level associations).

## Results

### Descriptive statistics, correlations, and dimensionality

Table 1 shows descriptive statistics and bivariate correlations. As expected, correlations showed that neuroticism was significantly negatively related to all self-control dimensions, while extraversion and conscientiousness was significantly positively related to all self-control dimensions. In line with expectations, agreeableness and openness to experience showed either weakly or unrelated relationships with all self-control dimensions. Facet level correlations are beyond the scope of focus for this paper, but the results are provided in the S1 Table.

**Table 1:** Means, standard deviations, and correlations with confidence intervals for personality traits and self-control.

Variable	*M*	*SD*	1	2	3	4	5	6	7
1. General self-control	3.77	0.35							
2. Inhibitory self-control	3.69	0.46	.85** [.82, .87]						
3. Initiatory self-control	3.86	0.41	.76** [.72, .80]	.31** [.22, .38]					
4. Neuroticism	2.39	0.38	-.32** [-.40, -.24]	-.34** [-.42, -.26]	-.15** [-.24, -.07]				
5. Extraversion	3.67	0.37	.28** [.19, .36]	.21** [.12, .29]	.23** [.14, .31]	-.25** [-.33, -.16]			
6. Openness to experience	3.40	0.42	.10* [.01, .19]	.11* [.02, .19]	.05 [-.04, .14]	-.04 [-.13, .05]	.37** [.29, .44]		
7. Agreeableness	3.54	0.36	.15** [.06, .23]	.17** [.08, .26]	.06 [-.03, .15]	-.23** [-.31, -.14]	.21** [.12, .29]	.37** [.29, .45]	
8. Conscientiousness	3.79	0.41	.37** [.29, .45]	.43** [.35, .50]	.14** [.05, .23]	-.51** [-.57, -.44]	.27** [.19, .35]	.15** [.06, .24]	.46** [.39, .53]

*N* = 480. *M* and *SD* are used to represent mean and standard deviation, respectively. Values in square brackets indicate the 95% confidence interval for each correlation. The confidence interval is a plausible range of population correlations that could have caused the sample correlation [80].

* indicates *p* < .05

** indicates *p* < .01.

A CFA showed that the MSCS had acceptable to good fit (CFI = .92; TLI = .91; RMSEA = .04; RMSEA 90% CI [.037, .048]; SRMR = .06), although the chi-square was significant, *X*^2^(362, *N* = 480) = 642.38, *p* < .001. These findings replicate previous research on the factor structure of the MSCS [16, 81].

### Unique effects

Table 2 displays the results from the linear multiple regression analysis that identified unique contributions of the individual personality traits to the self-control variables when controlling for other personality traits, sex, and age. Sex and age were not significant predictors of any self-control factors. Neuroticism and conscientiousness had significant unique effects on general self-control, while extraversion had not, *R*^*2*^ = .18, *F*(7, 472) = 14.77, *p* < .001. Conscientiousness and neuroticism had significant unique effects on inhibitory self-control, *R*^*2*^ = .20, *F*(7, 472) = 16.65, *p* < .001. Both conscientiousness and extraversion had significant unique effects on initiatory self-control, but neuroticism had not, *R*^*2*^ = .08, *F*(7, 472) = 5.49, *p* < .001. Neither openness to experience, nor agreeableness had any unique effects on any of the self-control dimensions. Given the insignificant unique effects of openness to experience and agreeableness on any self-control dimension, these traits were excluded from further analyses.

**Table 2:** Unique effects of sex, age and personality traits on self-control.

Predictor	General self-control			Inhibitory self-control			Initiatory self-control		
	*β*	*p*	95% CI	*β*	*p*	95% CI	*β*	*p*	95% CI
Sex	.08	.362	[-.093] [.254]	.03	.718	[-.136] [.198]	.11	.293	[-.092] [.303]
Age	-.02	.344	[-.053] [.019]	.01	.940	[-.033] [.036]	-.03	.126	[-.073] [.009]
Neuroticism	-.15	.001	[-.247] [-.060]	-.17	.001	[-.255] [-.075]	-.07	.172	[-.180] [.032]
Extraversion	.07	.091	[-.011] [.157]	-.01	.761	[-.094] [.069]	.15	.002	[.055] [.248]
Openness to experience	.04	.300	[-.038] [.124]	.02	.544	[-.054] [.102]	.05	.277	[-.041] [.143]
Agreeableness	-.02	.699	[-.104] [.070]	.01	.980	[-.085] [.083]	-.03	.564	[-.128] [.070]
Conscientiousness	.28	.001	[.182] [.367]	.31	.001	[.219] [.399]	.12	.026	[.014] [.226]

*N* = 480, CI = confidence interval.

### General self-control—the moderating effect of neuroticism

**Fig 1:**
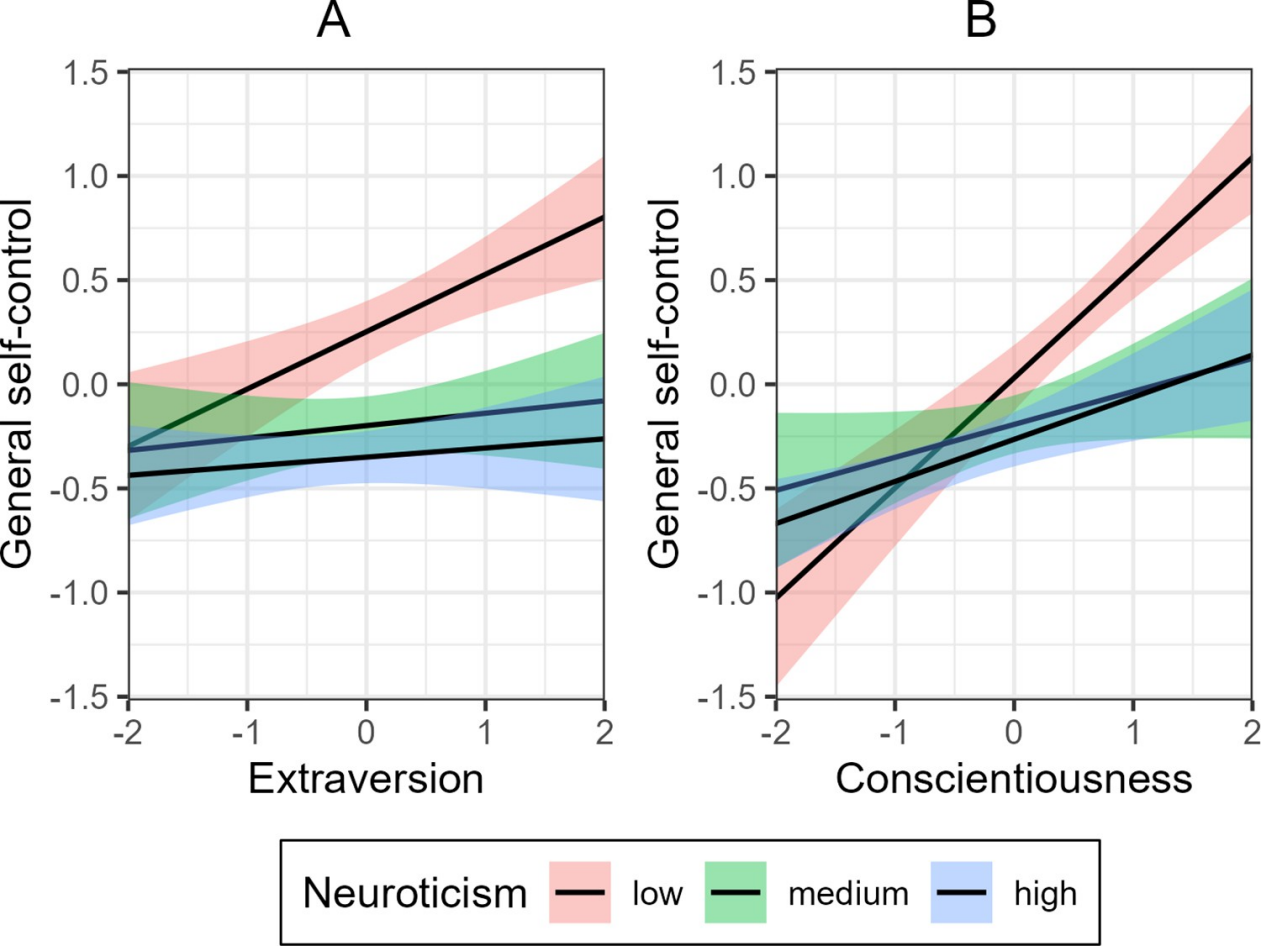
Two-way interactions for extraversion x neuroticism and conscientiousness x neuroticism on general self-control. Error bars denote 95% confidence intervals.

First, we examined to what extent neuroticism moderated the relationship between extraversion and general self-control. Table 3 and Fig 1A show a substantial and significant moderation for this relationship (*p <* .001). Individuals with low levels of neuroticism showed higher general self-control with increasing levels of extraversion, compared to individuals that had medium or high levels of neuroticism. The total ANOVA model that compared a model without and with interaction terms, was significant, *F*(2, 471) = 4.99, *p* = .007, thus favoring the more complex model. The regression analysis for the interaction model yielded an *R*^*2*^ of .19, *F*(8, 471) = 15.11, *p* < .001. Analyses of neuroticism facets revealed that anger hostility and self-consciousness significantly moderated the relationship between extraversion and general self-control (S1 Appendix).

**Table 3:** Main and moderation effects for extraversion x neuroticism and conscientiousness x neuroticism on self-control dimensions.

Predictor	General self-control			Inhibitory self-control			Initiatory self-control		
	*β*	*p*	95% CI	*β*	*p*	95% CI	*β*	*p*	95% CI
**Extraversion x neuroticism**									
E	.26	.001	[.126] [.389]	.19	.003	[.065] [.317]	.23	.003	[.083] [.387]
C	.28	.001	[.194] [.366]	.32	.001	[.239] [.404]	.11	.026	[.014] [.212]
E x N (m)	-.24	.014	[-.438] [-.051]	-.30	.002	[-.484] [-.112]	-.07	.532	[-.294] [.152]
E x N (h)	-.27	.004	[-.453] [-.089]	-.30	.001	[-.471] [-.122]	-.13	.239	[-.335] [.084]
**Conscientiousness x neuroticism**									
E	.09	.019	[.015] [.170]	-.01	.969	[-.077] [.074]	.17	.001	[.087] [.263]
C	.52	.001	[.367] [.677]	.44	.001	[.292] [.595]	.41	.001	[.233] [.587]
C x N (m)	-.39	.001	[-.625] [-.164]	-.21	.073	[-.430] [.020]	-.47	.001	[-.738] [-.212]
C x N (h)	-.34	.001	[-.540] [-.140]	.18	.071	[-.375] [.015]	-.41	.001	[-.635] [-.178]

*N* = 480, CI = confidence interval. Low Neuroticism was baseline value for all models. E = extraversion; O = openness to experience; A = agreeableness; C = conscientiousness; N = neuroticism; (m) = medium; (h) = high.

Then we examined to what extent neuroticism moderated the relationship between conscientiousness and general self-control. Table 3 and Fig 1B show that neuroticism moderated this relationship (*p* < .001): low levels of neuroticism strengthen the positive effect of conscientiousness on self-control compared to medium or high levels of neuroticism. The total ANOVA analysis favored the interaction model, *F*(2, 471) = 7.36, *p* < .001. Furthermore, the regression analysis for the interaction model yielded an *R*^*2*^ of .20, *F*(8, 471) = 15.84, *p* < .001. Examinations of neuroticism facets showed that anxiety, anger hostility, depression, impulsiveness, and vulnerability significantly moderated the relationship between conscientiousness and general self-control (S1 Appendix).

### Inhibitory and initiatory self-control–the moderating effect of neuroticism

**Fig 2:**
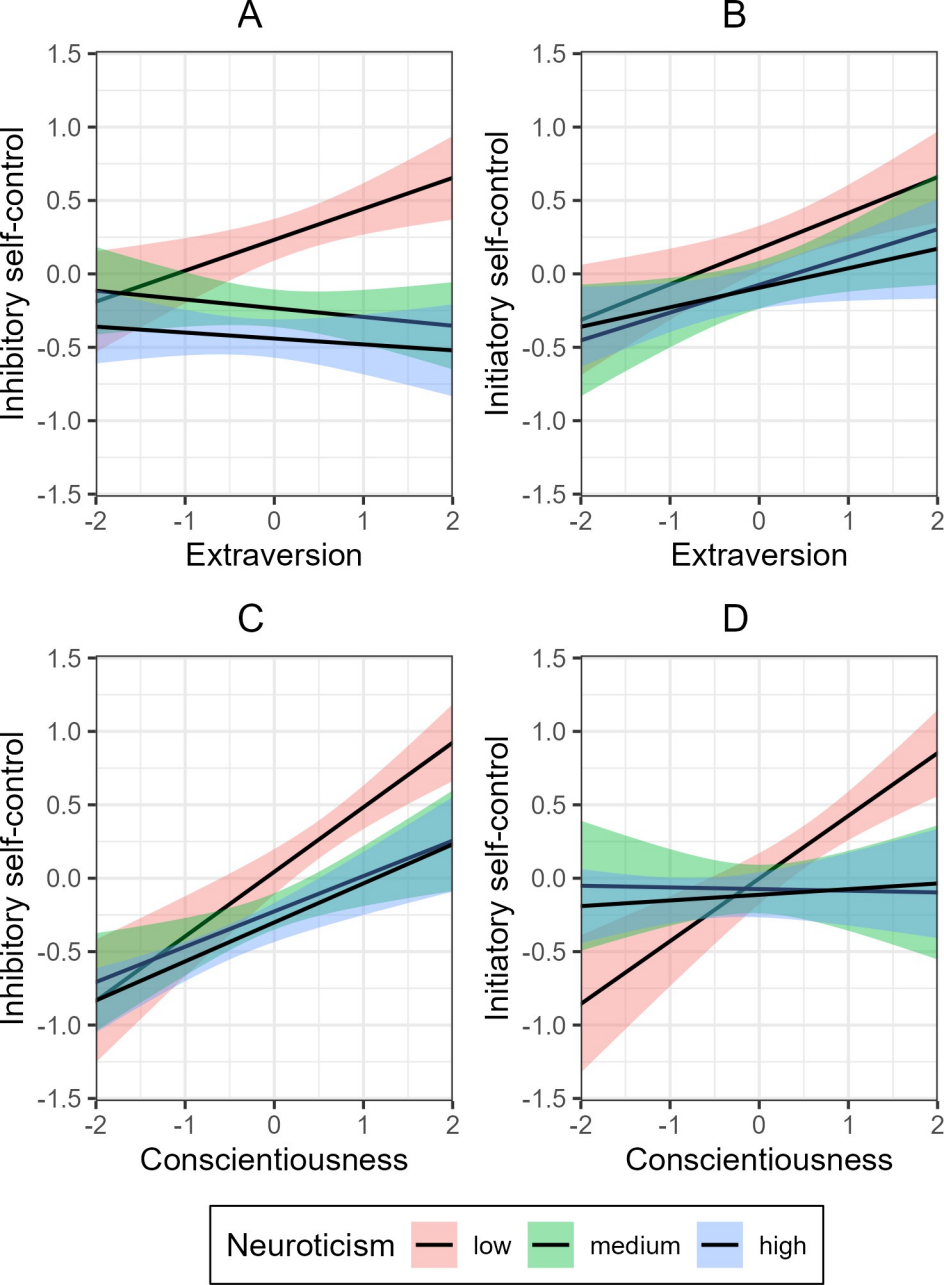
Two-way interaction for extraversion x neuroticism and conscientiousness x neuroticism on inhibitory and initiatory self-control. Error bars denote 95% confidence intervals.

We assessed to what extent neuroticism moderated the relationship between the two personality traits, extraversion and conscientiousness, on the one hand, and inhibitory and initiatory self-control on the other. As shown in Fig 2A and Table 3, neuroticism moderated the relationship between extraversion and *inhibitory* self-control (*p* < .001). This indicates that low levels of neuroticism strengthen the positive effect of extraversion on inhibitory self-control, compared to medium or high levels of neuroticism. A one-way ANOVA compared models with and without interactions terms, and favored the interaction model, *F*(2, 471) = 7.07, *p* < .001. The regression analysis with interaction terms yielded an *R*^2^ of .22, *F*(8, 471) = 17.53, *p* < .001. Investigations into neuroticism facets found that anger hostility significantly moderated the relationship between extraversion and inhibitory self-control (S1 Appendix).

We found no moderator effect of neuroticism on the relationship between extraversion and *initiatory* self-control (Fig 2B and Table 3), indicating that different levels of neuroticism do not significantly influence the relationship between extraversion and initiatory self-control. Nonetheless, upon closer examination at the facet level, we discovered that moderate levels of anger hostility significantly moderated the relationship between extraversion and initiatory self-control (S1 Appendix).

Fig 2C and Table 3 show that there was no moderation effect of neuroticism on the relationship between conscientiousness and *inhibitory* self-control, indicating that different levels of neuroticism do not significantly influence the relationship between conscientiousness and inhibition. However, when considering specific facets, we observed that heightened levels of depression and vulnerability had significant moderating effects on the relationship between conscientiousness and inhibitory self-control (S1 Appendix).

Fig 2D and Table 3 show that neuroticism moderated the relationship conscientiousness and *initiatory* self-control (*p* < .001). This indicate that low levels of neuroticism strengthen the positive effect of conscientiousness on initiatory self-control compared medium or high levels of neuroticism. The total ANOVA favored the interaction model, *F*(2, 471) = 8.15, *p* < .001. Moreover, the regression analysis for the interaction model yielded an *R*^2^ of .09, *F*(8, 471) = 7.09, *p* < .001. Through the analyses of neuroticism facets, it was discovered that anxiety, anger hostility, depression, and vulnerability played a significant moderating role in the relationship between conscientiousness and inhibitory self-control (S1 Appendix).

To check for robustness, we ran the all the moderation analyses for personality traits to also include openness to experience and agreeableness as main effect (see S2 Appendix). No interaction effect was found from these analyses, nor did the inclusion of openness to experience and agreeableness as predictors lead to any substantial changes for the interaction analyses.

## Discussion

The goal of this study was to investigate how personality traits relate to self-control by examining correlations, unique impacts, and the extent to which neuroticism moderated the relationship between extraversion and conscientiousness, respectively, and self-control.

Initially, we want to highlight two main points from our findings. First, we found that the relationship between personality and self-control depends on the aspect of self-control being examined–inhibitory or initiatory self-control. Secondly, we found that it is crucial to take the moderating effect of neuroticism into account when examining the relationship between the personality traits conscientiousness and extraversion on the one hand, and self-control on the other.

### The relationship between personality and self-control

Neuroticism correlated negatively with all self-control dimensions, which is in accordance with prior studies [20]. Furthermore, our results corroborate findings that neuroticism was more strongly and negatively correlated with inhibitory self-control, than with initiatory self-control [16, 33]. However, when controlling for the other personality traits, neuroticism was only significantly and negatively related to general and inhibitory self-control, and not to initiatory self-control. Hence, the higher the level of neuroticism, the more difficult it is to resist unwanted thoughts, feelings, and behavior in the face of temptations. On the other side, the level of neuroticism was not associated with the extent to which people exhibited proactive strategies to reach long-term goals when they anticipated temptations that conflicted with these goals.

Former studies have shown inconsistent results for the relationship between extraversion and self-control [2, 82]. We found positive correlations between extraversion and all self-control dimensions. However, when controlling for the other personality traits, extraversion only predicted initiatory self-control but not general and inhibitory self-control. Hence, higher levels of extraversion (other personality traits held constant), which includes having an energetic and active approach to the world [83], is associated with self-control strategies that are proactive (initiatory self-control) rather than reactive (inhibitory self-control). This supports de Vries and van Gelder [20] conclusion that the association between personality and self-control is dependent on conceptualizations of self-control.

In accordance with prior studies [i.e., 2, 16], conscientiousness correlated positively with all self-control dimensions. This pattern persisted when we controlled for the four other personality traits.

As expected, and in line with former studies [2, 16, 20, 33], openness to experience and agreeableness correlated positively, albeit weakly, with inhibitory self-control and general self-control, but not with initiatory self-control. However, when controlling for other personality traits, openness to experience and agreeableness showed no relationships to any of the self-control dimensions.

### Neuroticism as moderator in the relationship between personality traits and self-control

The results from our exploratory analysis of interactions showed that the strength of the relationship between conscientiousness and self-control, and between extraversion and self-control, depended on the level of neuroticism. Furthermore, when differentiating between inhibitory and initiatory self-control, we found two patterns.

The first pattern showed that neuroticism moderated the relationship between conscientiousness and *initiatory*, but not inhibitory self-control. The facet level analyses of neuroticism found that four neuroticism facets moderated the relationship between conscientiousness and initiatory self-control. This indicate that emotionally stable individuals, particularly those scoring *low* on anxiety, anger hostility, depression, and vulnerability may benefit more from increasing levels of conscientiousness when it comes to exhibiting proactive strategies to reach long-term goals, compared to conscientious but more emotionally unstable individuals.

Furthermore, the neuroticism/conscientiousness interaction indicate that when the level of conscientiousness is in the lower end, medium and high neuroticism can be beneficial for initiatory self-control. A way of interpreting this finding can be that conscientiousness is the primary personality predictor of initiatory self-control. However, neuroticism may also function as a driver for initiatory self-control, but only when the level of conscientiousness is in the low end. The vigilance from feelings of anxiety, vulnerability, and anger hostility may stimulate less conscientious, but highly neurotic, individuals to engage in initiatory self-control behaviors, like starting to exercise.

The second pattern was detected for extraversion: neuroticism moderated the relationship between extraversion and *inhibitory*, but not initiatory self-control. The analysis of neuroticism facets revealed that anger hostility, depression, and vulnerability played a moderating role in the relationship between extraversion and inhibitory self-control. Different levels of neuroticism were associated with a change in the direction of the relationship between extraversion and inhibitory self-control. People scoring low on neuroticism tended to benefit from increasing levels of extraversion when it came to showing inhibitory self-control, while people scoring medium or high on neuroticism, tended to show less inhibitory self-control with increasing levels of extraversion.

In essence, neuroticism appeared to be an important moderator for the relationship between conscientiousness and extraversion on the one hand, and self-control on the other, but with different effects depending on the type of self-control. This finding concerns prior discussions about the regulatory role of neuroticism as a control mechanism. It has been argued by the Carver model for self-control [41] that neuroticism [54, 84] acts as a breaking mechanism, while the UPPS-model argues that neuroticism decreases self-control [40]. Our results indicate that both arguments may be true, but under different conditions. In situations where conscientiousness is low, high neuroticism may be especially beneficial for initiatory self-control. However, in most cases, high (and often also medium) neuroticism is related to reduced inhibitory and initiatory self-control.

The relationship between self-control and emotions is well established. For instance, Heatherton et al. [55] have shown that people are aware that self-control is negatively related to negative emotions. Also, people seem to be aware of the importance of downregulating emotions when facing self-control challenges [85], and actually intend to do this in such situations [38, 86]. Our findings indicate that the neuroticism facets with the strongest association to negative affectivity, specifically anxiety, anger hostility, and vulnerability [87], exhibited the most pronounced moderation effects on self-control. In contrast, traits such as self-consciousness and impulsiveness, which have a weaker relationship to negative affectivity, showed fewer and weaker moderation effects and had insignificant ANOVA models. We suggest that the moderation effect indicated by levels of neuroticism in this study are in line with the *reactivity hypothesis*; the decreased ability of individuals with higher levels of neuroticism to downregulate negative emotions compared with individuals who score low on neuroticism [36, 88–90]. Javaras et al. [91] have shown that both self-control and conscientiousness are important for recovery from negative emotions. Our results inform this relationship further by showing that the neuroticism/conscientiousness interaction only predict changes in the initiatory component of self-control, and not the inhibitory component of self-control.

### Practical implications

As self-control can be a valuable resource both for good health, success, and proper conduct, knowledge about potential antecedents for self-control could be useful in applied settings. For instance, knowledge about strengths and weaknesses of personality profiles and their accompanying self-control qualities is helpful in selecting individuals for professions that require high self-control. In clinical and personal growth settings, the development and training of self-control may benefit from an increased understanding of the relationship between personality profiles and self-control patterns. For instance, clients who are high in neuroticism and extraversion may benefit from an awareness that they may be more likely to have challenges with inhibitory self-control, compared to those that have low neuroticism and high extraversion. On the other hand, the vigilance related to high neuroticism may be a resource for individuals who score low on conscientiousness.

### Limitations, strengths, and future directions

There are several limitations in our study. First, the sample consisted of mostly young male cadets and may not represent the general population. However, in another study on self-control [16], peers from a sample of military cadets (*n* = 948, 736 male, *M*_*age*_ = 19.8 years) were compared with a validation sample from the general population (*n* = 1331, 422 male, *M*_*age*_ = 50.3 years). The comparison showed that the associations between self-control and other variables did not differ between the two samples. Although the current study had a high participation rate and controlled for sex and age, generalizations of the results to other populations should still be done with caution and further studies in more demographically diverse populations are warranted.

When applying self-report measures there may be reasons for concerns about common source bias and common method bias [76, 92]. The data for personality and self-control were collected at two different occasions with 9 weeks in between, which might reduce commons source bias. A related limitation is that assessment of self-control and personality relied on self-report. A meta-analysis has shown that other-ratings of traits appear to be readily expressed to those acquainted with targets and that other-ratings are better than self-ratings in predicting performance-related behavior [93]. Furthermore, Siemsen et al. [94] have shown that analyses of interaction effects are particularly robust against common method bias. Even though our control for a methods factor did not indicate that any problematic results, there is a possibility that our findings can be inflated due to common method bias. We therefore recommend future studies to supplement self-ratings with other-ratings to increase the validity of the measurements.

## Conclusions

This study implies that it is important to differentiate between the types of self-control when studying the relationship between personality and self-control. The relationship between personality and self-control should not be studied without distinguishing between self-control as the ability to resist temptation (inhibitory self-control), and self-control as the ability to initiate proactive actions to achieve long-term goals (initiatory self-control). Furthermore, it is insufficient to study the direct effects of personality traits on self-control, and one needs to consider the interaction effects between personality traits and facets for their relationship with self-control. Neuroticism moderated the relationship between the two personality traits conscientiousness and extraversion, and self-control, and the interaction effects were different dependent on the type of self-control.

## Supporting information

S1 Table Bivariate correlations between personality and self-control. *Note*. *N* = 473. MSCS = Multi-Dimensional Self-Control Scale; INHIB = inhibition; INIT = initiation; BMSCS = Brief Multi-Dimensional Self-Control Scale; PRO = procrastination; IC = impulse control; AC = attentional control; EC = emotional control; GO = goal orientation; SCS = self-control strategies; Self-Cons. = self-conscientiousness; Exc. Seeking = excitement-seeking; Pos. Emotions = positive emotions; St.forwardness = straightforwardness; Tender-Mind. = tendermindedness; Ach. Striving = achievement striving. *p < .05. **p < .01.
(DOCX)

S1 Appendix Analyses for neuroticism facets as moderators. (DOCX)

S2 Appendix Moderation analyses for all personality traits. (DOCX)
