# Supplementary material for: Personality traits and self-control: The moderating role of neuroticism
Source: PLoS One. 2024 Aug 21;19(8):e0307871. doi: 10.1371/journal.pone.0307871 (PMC11338463; doi:10.1371/journal.pone.0307871)
Supplement: S1 Table — Note. N = 473. MSCS = Multi-Dimensional Self-Control Scale; INHIB = inhibition; INIT = initiation; BMSCS = Brief Multi-Dimensional Self-Control Scale; PRO = procrastination; IC = impulse control; AC = attentional control; EC = emotional control; GO = goal orientation; SCS = self-control strategies; Self-Cons. = self-conscientiousness; Exc. Seeking = excitement-seeking; Pos. Emotions = positive emotions; St.forwardness = straightforwardness; Tender-Mind. = tendermindedness; Ach. Striving = achievement striving. *p < .05. **p < .01. (DOCX) [file pone.0307871.s001.docx]

**S1 Table.** **Bivariate correlations between personality and self-control**

|  | MSCS | INHIB | INIT | BMSCS | PRO | AC | IC | EC | GO | SCS |
| --- | --- | --- | --- | --- | --- | --- | --- | --- | --- | --- |
| *Neuroticism* | *-.33^**^* | *-.35^**^* | *-.18^**^* | *-.22^**^* | *-.12^*^* | *-.30^**^* | *-.19^**^* | *-.15^**^* | *-.14^**^* | *-.08* |
| Anxiety | -.23^**^ | -.25^**^ | -.11^*^ | -.14^**^ | -.04 | -.25^**^ | -.14^**^ | -.12^*^ | -.09^*^ | -.03 |
| Angry hostility | -.34^**^ | -.35^**^ | -.18^**^ | -.21^**^ | -.14^**^ | -.28^**^ | -.21^**^ | -.16^**^ | -.15^**^ | -.08 |
| Depression | -.25^**^ | -.28^**^ | -.12^*^ | -.15^**^ | -.07 | -.26^**^ | -.15^**^ | -.10^*^ | -.09 | -.06 |
| Self-consciousness | -.25^**^ | -.24^**^ | -.16^**^ | -.17^**^ | -.08 | -.22^**^ | -.10^*^ | -.13^**^ | -.13^**^ | -.07 |
| Impulsiveness | -.28^**^ | -.27^**^ | -.17^**^ | -.19^**^ | -.15^**^ | -.18^**^ | -.17^**^ | -.13^**^ | -.13^**^ | -.09 |
| Vulnerability | -.27^**^ | -.30^**^ | -.14^**^ | -.21^**^ | -.08 | -.28^**^ | -.16^**^ | -.11^*^ | -.11^*^ | -.06 |
| *Extraversion* | *.28^**^* | *.21^**^* | *.25^**^* | *.23^**^* | *.15^**^* | *.22^**^* | *-.02* | *.22^**^* | *.11^*^* | *.21^**^* |
| Warmth | .26^**^ | .18^**^ | .24^**^ | .21^**^ | .14^**^ | .17^**^ | -.002 | .21^**^ | .10^*^ | .21^**^ |
| Gregariousness | .22^**^ | .19^**^ | .16^**^ | .16^**^ | .08 | .25^**^ | -.043 | .12^**^ | .08 | .15^**^ |
| Assertiveness | .27^**^ | .20^**^ | .24^**^ | .24^**^ | .16^**^ | .14^**^ | .065 | .19^**^ | .11^*^ | .21^**^ |
| Activity | .22^**^ | .15^**^ | .22^**^ | .15^**^ | .08 | .22^**^ | -.068 | .21^**^ | .10^*^ | .17^**^ |
| Excitement-seeking | .21^**^ | .13^**^ | .22^**^ | .20^**^ | .13^**^ | .14^**^ | -.046 | .16^**^ | .10^*^ | .22^**^ |
| Positive emotions | .19^**^ | .15^**^ | .16^**^ | .16^**^ | .11^*^ | .15^**^ | -.019 | .19^**^ | .07 | .09^*^ |
| *Openness to experience* | *.10^*^* | *.10^*^* | *.05* | *.092^*^* | *-.02* | *.13^**^* | *.037* | *.05* | *-.04* | *.11^*^* |
| Fantasy | .02 | .01 | .04 | .01 | -.070 | .06 | -.001 | .05 | -.07 | .10^*^ |
| Aesthetics | .06 | .06 | .03 | .08 | -.017 | .11^*^ | -.018 | .01 | -.02 | .09^*^ |
| Feelings | .14^**^ | .15^**^ | .07 | .12^**^ | .033 | .16^**^ | .067 | .08 | .01 | .08 |
| Actions | .03 | .05 | .01 | .06 | -.039 | .11^*^ | -.024 | .02 | -.07 | .06 |
| Ideas | .13^**^ | .17^**^ | .06 | .13^**^ | .005 | .16^**^ | .081 | .06 | -.02 | .10^*^ |
| Values | .11^*^ | .11^*^ | .06 | .08 | .023 | .08 | .107^*^ | .05 | -.01 | .10^*^ |
| *Agreeableness* | *.15^**^* | *.18^**^* | *.05* | *.12^**^* | *.086* | *.15^**^* | *.080* | *.13^**^* | *-.04* | *.03* |
| Trust | .17^**^ | .22^**^ | .03 | .14^**^ | .116^*^ | .19^**^ | .093^*^ | .05 | .01 | .02 |
| Straightforwardness | .10^*^ | .13^**^ | .03 | .03 | .027 | .14^**^ | .047 | .10^*^ | -.04 | .01 |
| Altruism | .11^*^ | .14^**^ | .02 | .10^*^ | .072 | .09 | .099^*^ | .08 | -.04 | .01 |
| Compliance | .09 | .14^**^ | -.02 | .08 | .054 | .16^**^ | .020 | .10^*^ | -.10^*^ | -.02 |
| Modesty | .12^*^ | .08 | .11^*^ | .13^**^ | .087 | .04 | .033 | .13^**^ | .04 | .09 |
| Tendermindedness | .12^*^ | .09 | .11^*^ | .11^*^ | .046 | .04 | .078 | .20^**^ | -.01 | .06 |
| *Conscientiousness* | *.38^**^* | *.43^**^* | *.15^**^* | *.33^**^* | *.26^**^* | *.31^**^* | *.22^**^* | *.11^*^* | *.18^**^* | *.03* |
| Competence | .33^**^ | .39^**^ | .12^**^ | .30^**^ | .24^**^ | .27^**^ | .20^**^ | .11^*^ | .14^**^ | .01 |
| Order | .32^**^ | .36^**^ | .14^**^ | .26^**^ | .21^**^ | .27^**^ | .17^**^ | .07 | .17^**^ | .05 |
| Dutifulness | .32^**^ | .37^**^ | .13^**^ | .29^**^ | .20^**^ | .27^**^ | .20^**^ | .13^**^ | .13^**^ | .01 |
| Achievement striving | .32^**^ | .39^**^ | .10^*^ | .27^**^ | .20^**^ | .32^**^ | .15^**^ | .08 | .13^**^ | .01 |
| Self-discipline | .33^**^ | .35^**^ | .16^**^ | .30^**^ | .20^**^ | .26^**^ | .17^**^ | .14^**^ | .17^**^ | .04 |
| Deliberation | .34^**^ | .39^**^ | .15^**^ | .31^**^ | .28^**^ | .22^**^ | .23^**^ | .03 | .22^**^ | .04 |

*Note.* *N* = 473. MSCS = Multi-Dimensional Self-Control Scale; INHIB = inhibition; INIT = initiation; BMSCS = Brief Multi-

Dimensional Self-Control Scale; PRO = procrastination; IC = impulse control; AC = attentional control; EC = emotional control;

GO = goal orientation; SCS = self-control strategies. **p* < .05. ***p* < .01.
