# Supplementary material for: Personality traits and self-control: The moderating role of neuroticism
Source: PLoS One. 2024 Aug 21;19(8):e0307871. doi: 10.1371/journal.pone.0307871 (PMC11338463; doi:10.1371/journal.pone.0307871)
Supplement: S1 Appendix — (DOCX) [file pone.0307871.s002.docx]

**Appendix S1: Analyses for neuroticism facets as moderators**

This appendix shows the results from analyses of whether any of the six facets of neuroticism moderated the relationship between the personality traits extraversion or conscientiousness, on one hand, and the three self-control dimensions on the other hand.

**General self-control - the moderating effect of neuroticism facets**

First, our investigation focused on examining the extent to which facets of neuroticism moderated the relationship between extraversion and general self-control. Table A and Figs A-F revealed that two out of six neuroticism facets significantly moderated these relationships. Specifically, individuals with low levels of anger hostility or self-consciousness exhibited higher general self-control with increasing levels of extraversion, in comparison to individuals with medium or high levels of these facets. The total ANOVA models, which compared a model without and with interaction terms, were found to be significant (anger hostility: *F*(2, 471) = 4.63, *p* = .010; self-consciousness: *F* (2, 471) = 3.37, *p* = .035), favoring the more complex model. The regression analysis for the anger hostility interaction model resulted in an *R^2^* of .18, *F* (8, 471) = 14.39, *p* < .001, while the regression analysis for the self-consciousness interaction model yielded an *R^2^* of .17, *F*(8, 471) = 13.33, *p* < .001.

**Table A. Main and moderation effects for neuroticism facets with extraversion and conscientiousness on general, inhibitory, and initiatory self-control.**

| **Predictor** |  | **General self-control** | | | |  | **Inhibitory self-control** | | | |  | **Initiatory self-control** | | | |  |
| --- | --- | --- | --- | --- | --- | --- | --- | --- | --- | --- | --- | --- | --- | --- | --- | --- |
|  |  | ***β*** | ***p*** | **95% CI** | |  | ***β*** | ***p*** | **95% CI** | |  | ***β*** | ***p*** | **95% CI** | |  |
| **Extraversion x anxiety** | | | | | | | | | | | | | | | | |
| E |  | .20 | .007 | [.055] | [.339] |  | .19 | .086 | [-.017] | [.256] |  | .21 | .009 | [.053] | [.375] |  |
| C |  | .32 | .001 | [.232] | [.399] |  | .36 | .001 | [.281] | [.442] |  | .13 | .008 | [.033] | [.223] |  |
| E x anxiety (m) |  | -.13 | .205^¶^ | [-.322] | [.069] |  | -.17 | .077^¶^ | [-.359] | [.018] |  | -.02 | .868 | [-.241] | [.203] |  |
| E x anxiety (h) |  | -.15 | .122^¶^ | [-.340] | [.040] |  | -.14 | .123^¶^ | [-.327] | [.039] |  | -.09 | .389 | [-.310] | [.121] |  |
| **Conscientiousness x anxiety** | | | | | | | | | | | | | | | |  |
| E |  | .10 | .012 | [.022] | [.179] |  | .01 | .868 | [-.069] | [.082] |  | .18 | .001 | [.091] | [.268] |  |
| C |  | .43 | .001 | [.286] | [.579] |  | .36 | .001 | [.219] | [.503] |  | .35 | .001 | [.180] | [.511] |  |
| C x anxiety (m) |  | -.02 | .842^¶^ | [-.235] | [.192] |  | .16 | .130 | [-.074] | [.365] |  | -.24 | .054^¶^ | [-.480] | [.004] |  |
| C x anxiety (h) |  | -.28 | .005^¶^ | [-.475] | [-.087] |  | -.12 | .215 | [-.306] | [.069] |  | -.37 | .001^¶^ | [-.592] | [-.153] |  |
| **Extraversion x anger hostility** | | | | | | | | | | | | | | | |  |
| E |  | .30 | .001 | [.146] | [.463] |  | .21 | .006 | [.062] | [.368] |  | .29 | .002 | [.110] | [.470] |  |
| C |  | .28 | .001 | [.199] | [.371] |  | .33 | .001 | [.251] | [.417] |  | .11 | .032 | [.009] | [.204] |  |
| E x anger h. (m) |  | -.30 | .003^¶^ | [-.495] | [-.101] |  | -.23 | .018^¶^ | [-.420] | [-.039] |  | -.26 | .026 | [-.479] | [-.031] |  |
| E x anger h. (h) |  | -.24 | .020^¶^ | [-.448] | [-.038] |  | -.34 | .001^¶^ | [-.533] | [-.137] |  | -.02 | .844 | [-.256] | [.210] |  |
| **Conscientiousness x angry hostility** | | | | | | | | | | | | | | | |  |
| E |  | .10 | .017 | [.017] | [.174] |  | .01 | .917 | [-.072] | [.080] |  | .17 | .001 | [.085] | [.263] |  |
| C |  | .44 | .001 | [.264] | [.613] |  | .41 | .001 | [.235] | [.574] |  | .31 | .003 | [.108] | [.504] |  |
| C x anger h. (m) |  | -.15 | .191^¶^ | [-.378] | [.075] |  | -.02 | .878 | [-.237] | [.203] |  | -.26 | .048^¶^ | [-.516] | [-.002] |  |
| C x anger h. (h) |  | -.24 | .030^¶^ | [-.461] | [-.024] |  | -.16 | .138 | [-.373] | [.052] |  | -.25 | .045^¶^ | [-.502] | [-.006] |  |
| **Extraversion x depression** | | | | | | | | | | | | | | | |  |
| E |  | .11 | .125 | [-.031] | [.250] |  | -.01 | .961 | [-.139] | [.132] |  | .21 | .010 | [.051] | [.368] |  |
| C |  | .32 | .001 | [.235] | [.403] |  | .36 | .001 | [.282] | [.444] |  | .13 | .048 | [.039] | [.229] |  |
| E x depression (m) |  | .01 | .966^¶^ | [-.187] | [.195] |  | .07 | .480^¶^ | [-.118] | [.251] |  | -.07 | .501 | [-.290] | [.142] |  |
| E x depression (h) |  | -.03 | .759^¶^ | [-.223] | [.163] |  | -.03 | .786^¶^ | [-.212] | [.160] |  | -.03 | .805 | [-.246] | [.191] |  |
| **Conscientiousness x depression** | | | | | | | | | | | | | | | |  |
| E |  | .11 | .040 | [.032] | [.189] |  | .02 | .629 | [-.057] | [.095] |  | .18 | .001 | [.093] | [.271] |  |
| C |  | .49 | .001 | [.340] | [.637] |  | .50 | .001 | [.355] | [.643] |  | .28 | .001 | [.107] | [.445] |  |
| C x depression (m) |  | -.14 | .208^¶^ | [-.353] | [.077] |  | -.11 | .293 | [-.320] | [.097] |  | -.12 | .351^¶^ | [-.360] | [.128] |  |
| C x depression (h) |  | -.31 | .002^¶^ | [-.507] | [-.119] |  | -.25 | .010 | [-.436] | [-.060] |  | -.27 | .017^¶^ | [-.488] | [-.047] |  |

| **Predictor** |  | **General Self-Control** | | | |  | **Inhibitory Self-Control** | | | |  | **Initiatory Self-Control** | | | |  |
| --- | --- | --- | --- | --- | --- | --- | --- | --- | --- | --- | --- | --- | --- | --- | --- | --- |
|  |  | ***β*** | ***p*** | **95% CI** | |  | ***Β*** | ***p*** | **95% CI** | |  | ***β*** | ***p*** | **95% CI** | |  |
| **Extraversion x self-consciousness** | | | | | | | | | | | | | | | | |
| E |  | .23 | .007 | [.063] | [.390] |  | .13 | .122 | [-.034] | [.283] |  | .26 | .006 | [.074] | [.446] |  |
| C |  | .31 | .001 | [.229] | [.395] |  | .36 | .001 | [.280] | [.441] |  | .12 | .011 | [.028] | [.218] |  |
| E x self-cons. (m) |  | -.08 | .437^¶^ | [-.288] | [.125] |  | -.08 | .463^¶^ | [-.274] | [.125] |  | -.05 | .688 | [-.283] | [.187] |  |
| E x self-cons. (h) |  | -.25 | .017^¶^ | [-.450] | [-.044] |  | -.22 | .028^¶^ | [-.416] | [-.023] |  | -.18 | .131 | [-.408] | [.053] |  |
| **Conscientiousness x self-consciousness** | | | | | | | | | | | | | | | |  |
| E |  | .10 | .013 | [.021] | [.178] |  | .01 | .795 | [-.066] | [.086] |  | .17 | .001 | [.085] | [.263] |  |
| C |  | .37 | .001 | [.182] | [.553] |  | .37 | .001 | [.194] | [.552] |  | .22 | .042 | [.008] | [.428] |  |
| C x self-cons. (m) |  | .01 | .117^¶^ | [-.227] | [.232] |  | .07 | .520 | [-.149] | [.295] |  | -.09 | .493^¶^ | [-.352] | [.170] |  |
| C x self-cons. (h) |  | -.15 | .194^¶^ | [-.371] | [.076] |  | -.10 | .342 | [-.320] | [.111] |  | -.16 | .231^¶^ | [-.408] | [.099] |  |
| **Extraversion x impulsiveness** | | | | | | | | | | | | | | | |  |
| E |  | .13 | .103 | [-.027] | [.293] |  | .14 | .074 | [-.014] | [.295] |  | .07 | .452 | [-.112] | [.251] |  |
| C |  | .32 | .001 | [.235] | [.402] |  | .37 | .001 | [.290] | [.451] |  | .12 | .011 | [.028] | [.216] |  |
| E x impulsiveness (m) |  | .01 | .917^¶^ | [-.200] | [.222] |  | -.12 | .262^¶^ | [-.320] | [.087] |  | .17 | .167 | [-.071] | [.407] |  |
| E x impulsiveness (h) |  | -.11 | .265^¶^ | [-.312] | [.086] |  | -.23 | .019^¶^ | [-.424] | [-.039] |  | .09 | .461 | [-.141] | [.310] |  |
| **Conscientiousness x impulsiveness** | | | | | | | | | | | | | | | |  |
| E |  | .10 | .019 | [.016] | [.175] |  | .01 | .822 | [-.068] | [.086] |  | .17 | .001 | [.077] | [.258] |  |
| C |  | .46 | .001 | [.283] | [.633] |  | .48 | .001 | [.309] | [.649] |  | .24 | .017 | [.044] | [.441] |  |
| C x impulsiveness (m) |  | -.09 | .469^¶^ | [-.315] | [.146] |  | -.07 | .531 | [-.294] | [.152] |  | -.07 | .626^¶^ | [-.325] | [.196] |  |
| C x impulsiveness (h) |  | -.25 | .020^¶^ | [-.461] | [-.040] |  | -.20 | .058 | [-.402] | [.006] |  | -.22 | .077^¶^ | [-.453] | [.024] |  |
| **Extraversion x vulnerability** | | | | | | | | | | | | | | | |  |
| E |  | .17 | .019 | [.027] | [.305] |  | .12 | .093 | [-.019] | [.248] |  | .16 | .042 | [.006] | [.321] |  |
| C |  | .30 | .001 | [.212] | [.386] |  | .34 | .001 | [.260] | [.427] |  | .12 | .016 | [.022] | [.219] |  |
| E x vulnerability (m) |  | -.03 | .771^¶^ | [-.231] | [.171] |  | -.15 | .128^¶^ | [-.344] | [.044] |  | .13 | .250 | [-.094] | [.362] |  |
| E x vulnerability (h) |  | -.15 | .112^¶^ | [-.334] | [.035] |  | .16 | .071^¶^ | [-.341] | [.014] |  | -.07 | .514 | [-.279] | [.140] |  |
| **Conscientiousness x vulnerability** | | | | | | | | | | | | | | | |  |
| E |  | .10 | .009 | [.027] | [.182] |  | .01 | .755 | [-.063] | [.087] |  | .18 | .001 | [.090] | [.268] |  |
| C |  | .48 | .001 | [.310] | [.641] |  | .48 | .001 | [.325] | [.644] |  | .27 | .005 | [.080] | [.461] |  |
| C x vulnerability (m) |  | -.04 | .759^¶^ | [-.275] | [.201] |  | .02 | .879 | [-.212] | [.248] |  | -.09 | .504^¶^ | [-.366] | [.180] |  |
| C x vulnerability (h) |  | -.35 | .001^¶^ | [-.554] | [-.145] |  | -.31 | .002 | [-.506] | [-.111] |  | -.26 | .031^¶^ | [-.494] | [-.024] |  |

Note. *N* = 480, CI = confidence interval. Low levels of the neuroticism facet were the baseline values for all models. E = Extraversion; C = Conscientiousness; anger h. = anger hostility; self-cons. = self-consciousness; (m) = medium; (h) = high; ^¶^ = significant relationship at the factor level using N as a moderator (see Table 3).

Next, our investigation aimed to assess the extent to which facets of neuroticism moderated the relationship between conscientiousness and general self-control. It was found that five out of six neuroticism facets significantly moderated these relationships (Table A and Figs A-F). Specifically, individuals with low levels of anxiety, anger hostility, depression, impulsiveness, and vulnerability displayed higher general self-control as conscientiousness levels increased, in comparison to individuals with medium or high levels of these facets. The total ANOVA models were found to be significant for anxiety, *F*(2, 471) = 5.18, *p* = .006; depression, *F*(2, 471) = 7.31, *p* = .007; impulsiveness, *F*(2, 471) = 3.20, *p* = .042; and vulnerability, *F*(2, 471) = 7.50, *p* = .001. However, the ANOVA model for anger hostility was not significant, *F*(2, 471) = 2.37, *p* = .095. The regression analysis for the interaction models yielded the following results: anxiety, *R^2^*of .16, *F*(8, 471) = 12.53, *p* < .001; anger hostility, *R^2^* of .18, *F*(8, 471) = 13.71, *p* < .001; depression, *R^2^* of .17, *F*(8, 471) = 13.29, *p* < .001; impulsiveness, *R^2^* of .17, *F*(8, 471) = 12.98, *p* < .001; and vulnerability, *R^2^* of .19, F(8, 471) = 14.63, *p* < .001.

**Inhibitory self-control – the moderating effect of neuroticism facets**

We then explored the extent to which neuroticism facets moderated the relationship between extraversion and inhibitory self-control. As depicted in Table A and Figs A-F, three out of six neuroticism facets significantly influenced these relationships. Specifically, individuals with low levels of anger hostility, self-consciousness, or impulsiveness exhibited higher inhibitory self-control as extraversion levels increased, in contrast to individuals with medium or high levels of these facets. The ANOVA models that compared a model without and with interaction terms were significant for anger hostility, *F*(2, 471) = 7.34, *p* = .004, though not for self-consciousness, *F*(2, 471) = 3.73, *p* = .006, and impulsiveness *F*(2, 471) = 3.84, *p* = .006. The regression analysis for the interaction models yielded the following results: anger hostility, *R^2^* of .20, *F*(8, 471) = 15.67, *p* < .001; self-consciousness, *R^2^* of .18, *F*(8, 471) = 14.37, *p* < .001; and impulsiveness, *R^2^* of .18, *F*(8, 471) = 13.88, *p* < .001.

The investigation of the extent to which neuroticism facets moderated the relationship between conscientiousness and inhibitory self-control showed that two out of six neuroticism facets significantly influenced these relationships (Table A and Figs A-F). Specifically, individuals with low levels of depression or vulnerability displayed higher inhibitory self-control as conscientiousness levels increased, compared to individuals with medium or high levels of these facets. The total ANOVA models were significant for depression, *F*(2, 471) = 3.43, *p* = .033, and vulnerability, *F*(2, 471) = 7.37, *p* = .001. The regression analysis for the interaction models resulted in the following outcomes: depression, *R^2^* of .18, *F*(8, 471) = 14.18, *p* < .001; and vulnerability, *R^2^* of .20, *F*(8, 471) = 16.29, *p* < .001.

**Initiatory self-control – the moderating effect of neuroticism facets**

Our investigation centered next on exploring the extent to which neuroticism facets moderated the relationship between extraversion and initiatory self-control. Our findings, as demonstrated in Table A and Figs A-F, indicated that one out of the six neuroticism facets significantly influenced these relationships. Specifically, individuals with low levels of anger hostility exhibited higher initiatory self-control as levels of extraversion increased, in contrast to individuals with medium or high levels of this facet. The total ANOVA model for anger hostility was significant, *F*(2, 471) = 3.62, *p* = .028, and the regression analysis for the interaction model revealed an *R^2^* at .08, *F*(8, 471) = 6.32, *p* < .001.

Finally, our study investigated the extent to which neuroticism facets moderated the relationship between conscientiousness and initiatory self-control. Table A and Figs A-F revealed that four out of six neuroticism facets significantly influenced these relationships. Particularly, individuals with low levels of anxiety, anger hostility, depression, or vulnerability demonstrated higher initiatory self-control with increasing levels of conscientiousness, compared to individuals with medium or high levels of these facets. The total ANOVA models comparing a model without and with interaction terms were significant for anxiety, *F*(2, 471) = 5.60, *p* = .004, but not for anger hostility, *F*(2, 471) = 2.47, *p* = .085, depression, *F*(2, 471) = 2.93, *p* = .054, and vulnerability, *F*(2, 471) = 2.57, *p* = .078. The regression analysis for the interaction models yielded the following results: anxiety, *R^2^* of .08, *F*(8, 471) = 6.07, *p* < .001; anger hostility, *R^2^* of .07, *F*(8, 471) = 6.01, *p* < .001; depression, *R^2^* of .07, *F*(8, 471) = 5.29, *p* < .001; and vulnerability, *R^2^* of .07, *F*(8, 471) = 5.27, *p* < .001.

**Fig A. Two-way interactions for extraversion x anxiety and conscientiousness x anxiety on general, inhibitory, and initiatory self-control.
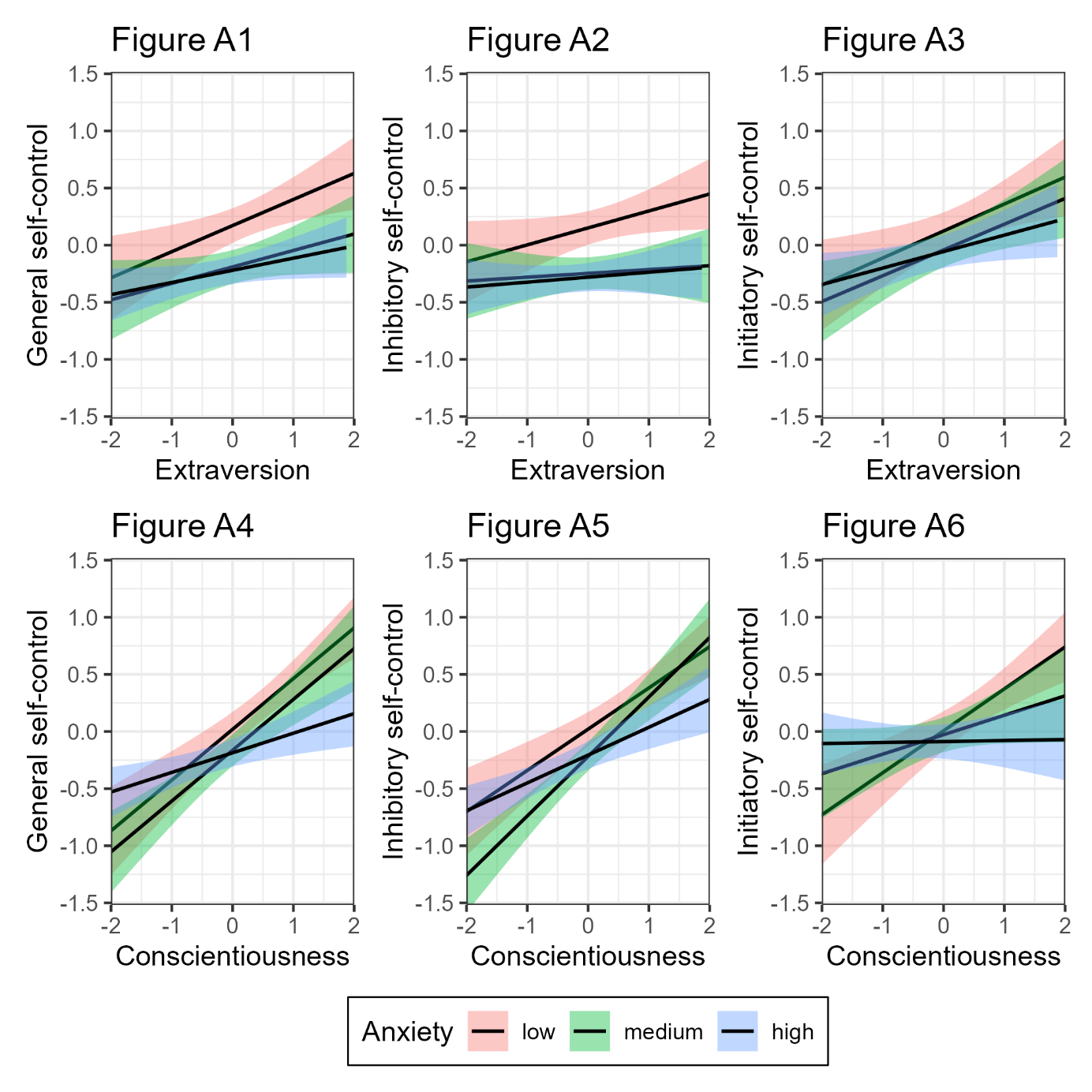
**

Error bars denote 95% confidence intervals.

**Fig B. Two-way interactions for extraversion x anger hostility and conscientiousness x anger hostility on general, inhibitory, and initiatory self-control.
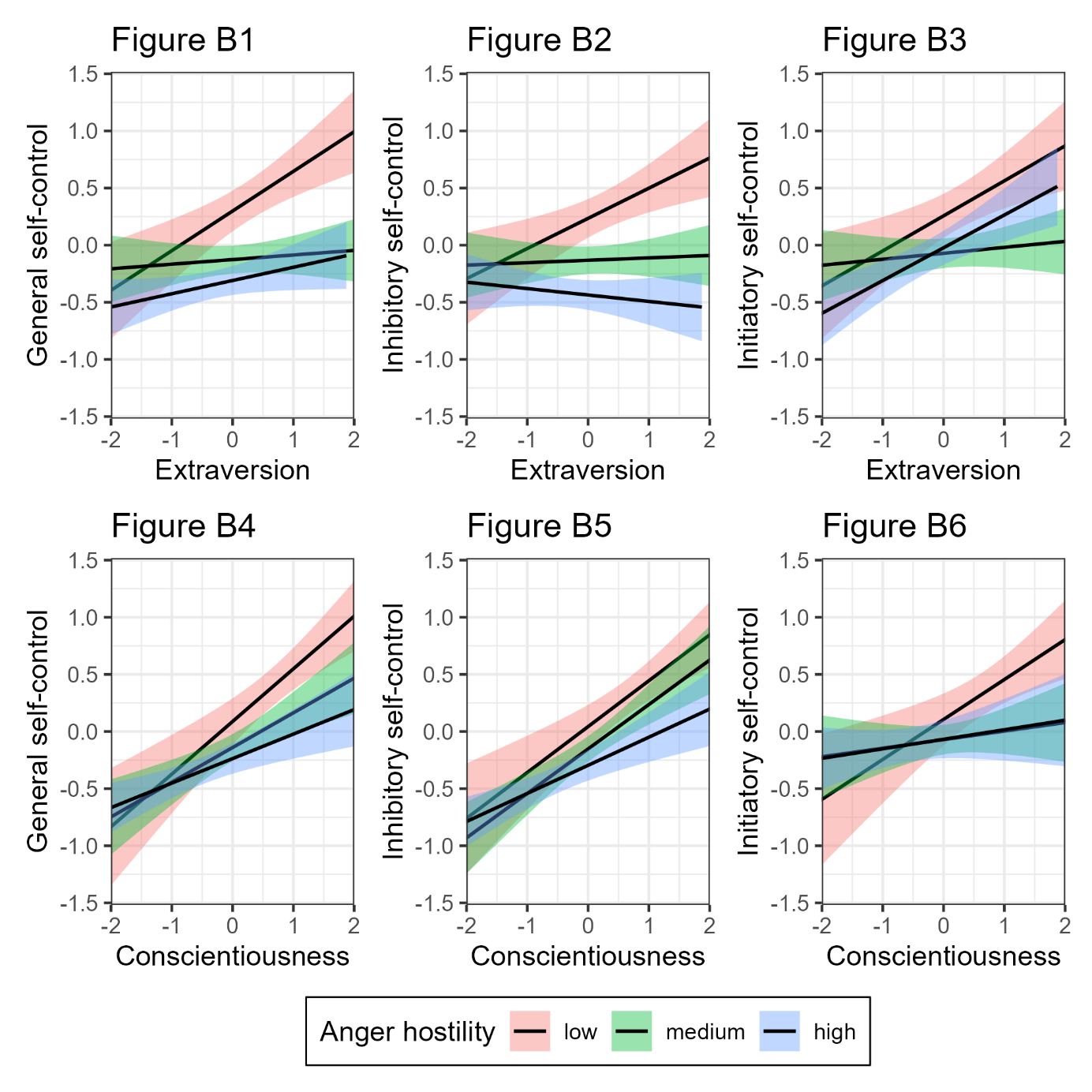
**

Error bars denote 95% confidence intervals.

**Fig C. Two-way interactions for extraversion x depression and conscientiousness x depression on general, inhibitory, and initiatory self-control.
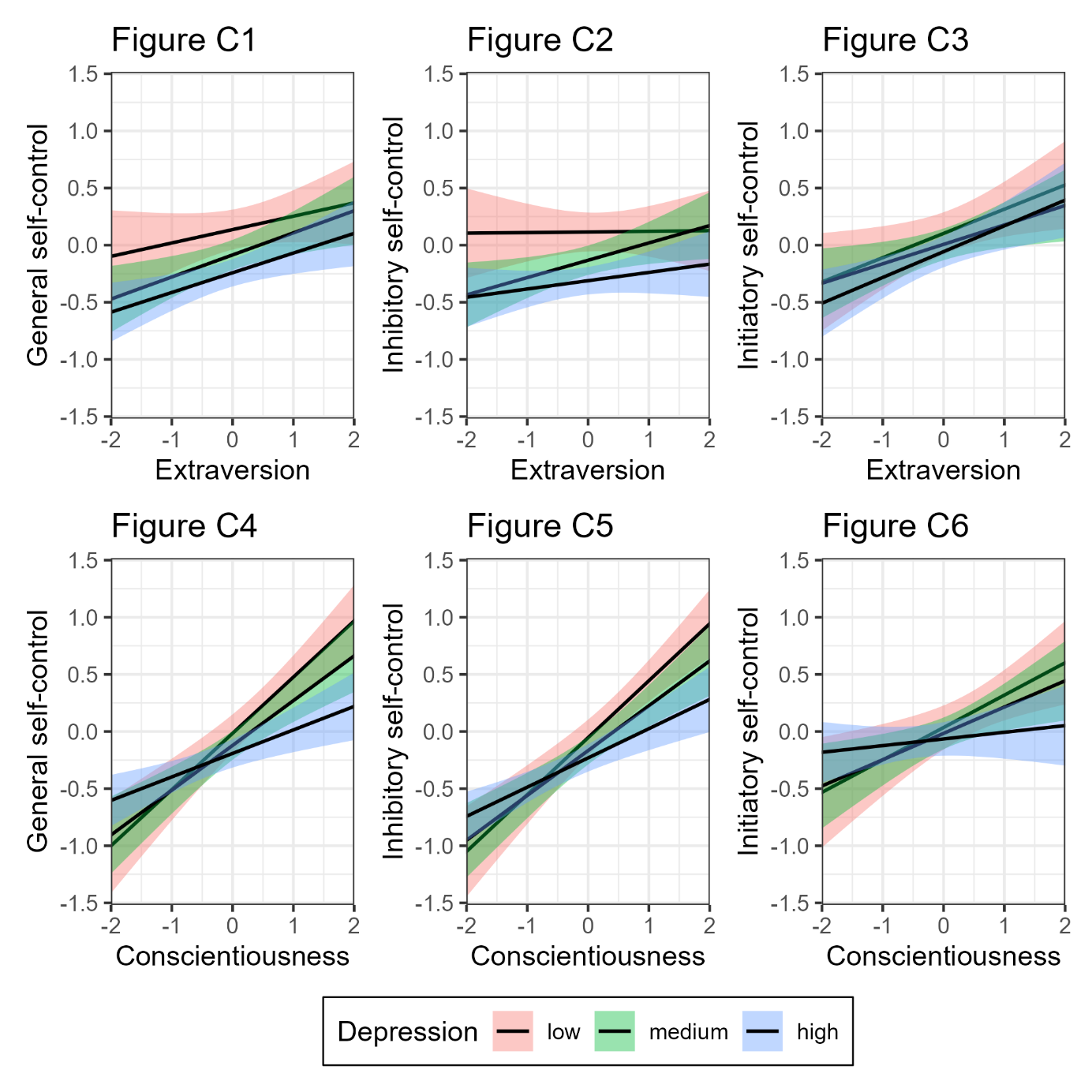
**

Error bars denote 95% confidence intervals.

**Fig D. Two-way interactions for extraversion x self-consciousness and conscientiousness x self-consciousness on general, inhibitory, and initiatory self-control.
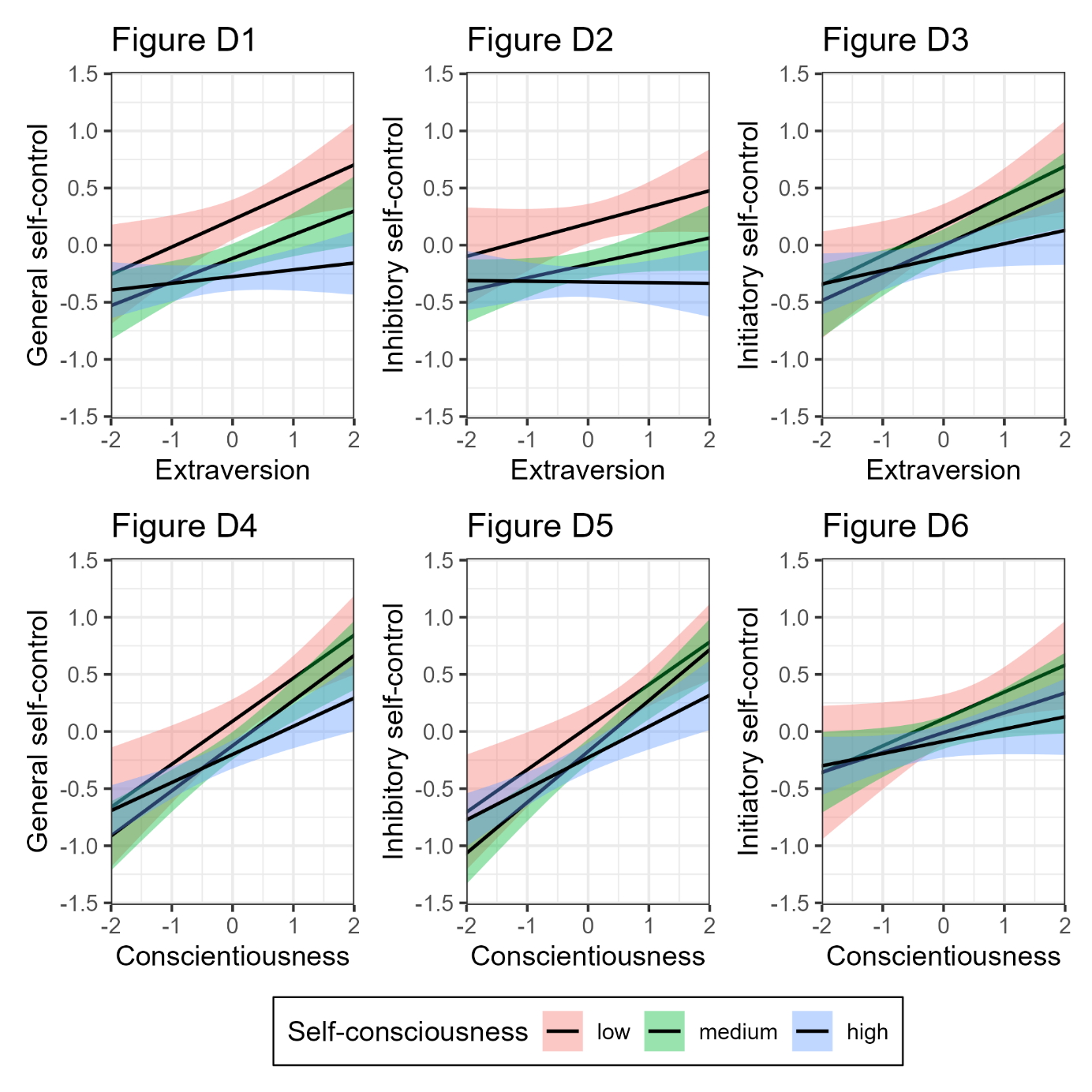
**

Error bars denote 95% confidence intervals.

**Fig E. Two-way interactions for extraversion x impulsiveness and conscientiousness x impulsiveness on general, inhibitory, and initiatory self-control.
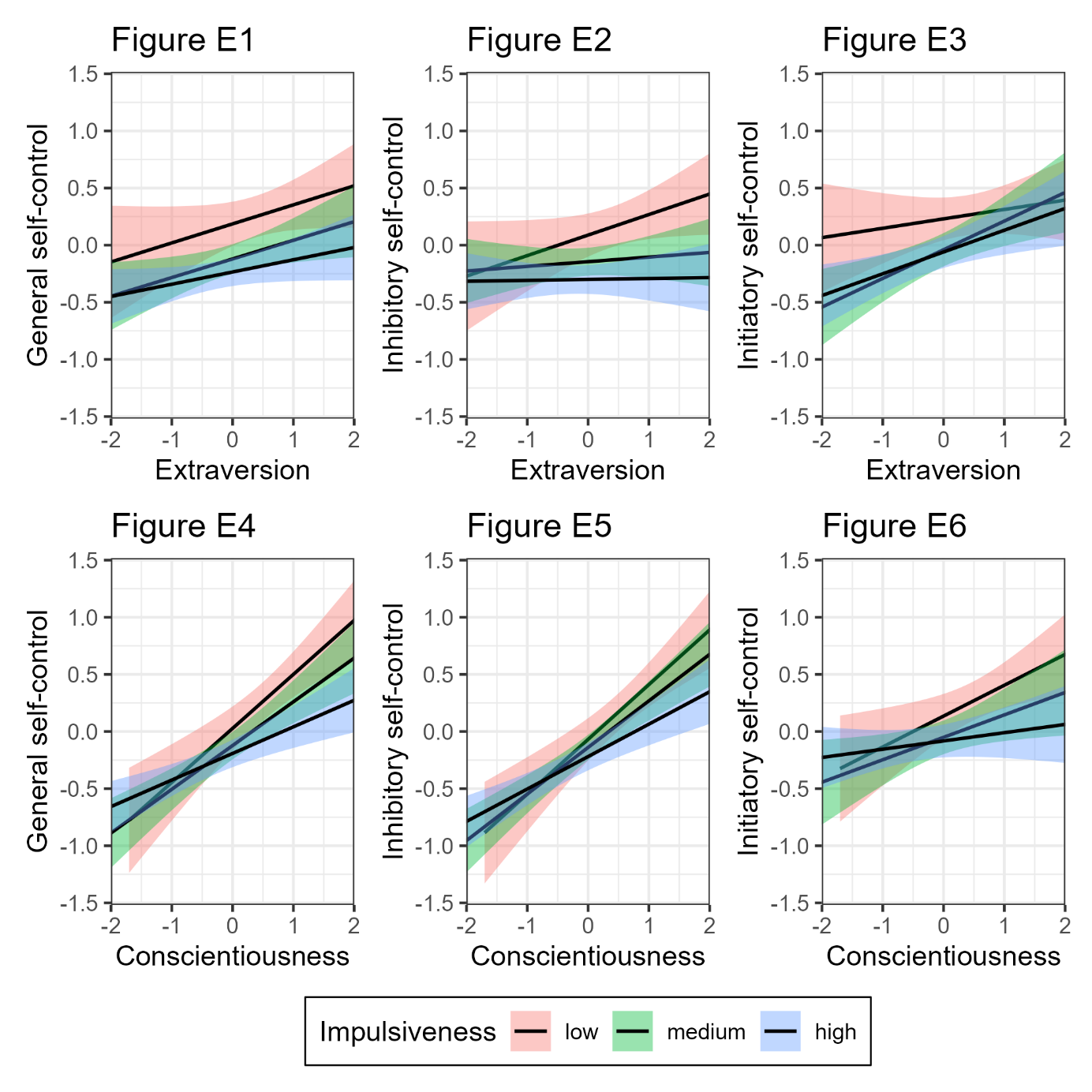
**

Error bars denote 95% confidence intervals.

**Fig F. Two-way interactions for extraversion x vulnerability and conscientiousness x vulnerability on general, inhibitory, and initiatory self-control.
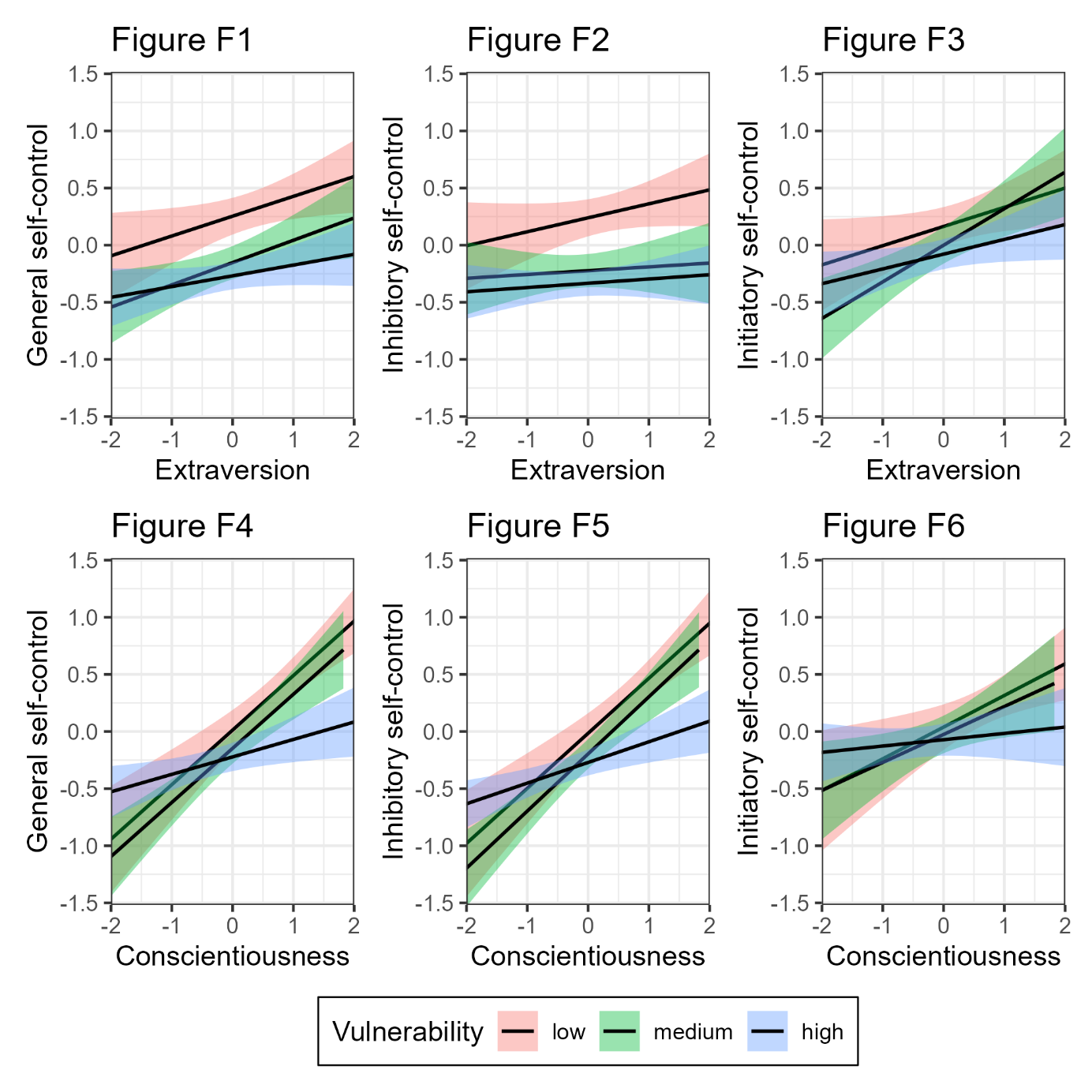
**

Error bars denote 95% confidence intervals.
