# Supplementary material for: Personality traits and self-control: The moderating role of neuroticism
Source: PLoS One. 2024 Aug 21;19(8):e0307871. doi: 10.1371/journal.pone.0307871 (PMC11338463; doi:10.1371/journal.pone.0307871)
Supplement: S2 Appendix — (DOCX) [file pone.0307871.s003.docx]

**Appendix S2: Moderation analyses for all personality traits**

This appendix serves as a robustness check for the results in the main article, presenting analyses of how neuroticism moderates the relationship between the personality traits and self-control when all four remaining personality traits of the Five-Factor Model are included.

**General self-control – the moderating effect of neuroticism**

First, we examined to what extent neuroticism moderated the relationship between extraversion and general self-control. As in the main article, Table B show a substantial and significant moderation for this relationship (*p <* .001). The total ANOVA model that compared a model without and with interaction terms, was significant, *F*(2, 469) = 4.96, *p* = .007, thus favoring the more complex model. The regression analysis for the interaction model yielded an *R^2^* of .19, *F*(10, 469) = 12.15, *p* < .001.

Next, we analyzed to what extent neuroticism moderated the relationship between openness to experience and general self-control, as well as the relationship between agreeableness and general self-control. Table C show that there were no interaction effects for these relationships.

Then we examined to what extent neuroticism moderated the relationship between conscientiousness and general self-control. As in the main article, Table B show that neuroticism moderated this relationship (*p* < .001). The total ANOVA analysis favored the interaction model, *F*(2, 469) = 7.19, *p* < .001. Furthermore, the regression analysis for the interaction model yielded an *R^2^* of .20, *F*(10, 469) = 12.70, *p* < .001.

**Table B. Main and moderation effects for extraversion x neuroticism and conscientiousness x neuroticism on general, inhibitory, and initiatory self-control.**

| Predictor |  | General self-control | | | |  | Inhibitory self-control | | | |  | Initiatory self-control | | | |  |
| --- | --- | --- | --- | --- | --- | --- | --- | --- | --- | --- | --- | --- | --- | --- | --- | --- |
|  |  | *β* | *p* | 95% CI | |  | *β* | *P* | 95% CI | |  | *β* | *p* | 95% CI | |  |
| Extraversion x neuroticism | | | | | | | | | | | | | | | | |
| E |  | .24 | .001 | [.108] | [.379] |  | .18 | .006 | [.053] | [.314] |  | .22 | .006 | [.062] | [.374] |  |
| O |  | .04 | .353 | [-.042] | [.118] |  | .02 | .646 | [-.059] | [.095] |  | .05 | .287 | [-.042] | [.142] |  |
| A |  | -.01 | .733 | [-.100] | [.071] |  | .01 | .947 | [-.078] | [.085] |  | -.03 | .552 | [-.129] | [.069] |  |
| C |  | .29 | .001 | [.197] | [.377] |  | .32 | .001 | [.236] | [.408] |  | .12 | .018 | [.021] | [.228] |  |
| E x N (m) |  | -.24 | .015 | [-.435] | [-.047] |  | -.30 | .002 | [-.483] | [-.111] |  | -.07 | .562 | [-.290] | [.158] |  |
| E x N (h) |  | -.27 | .003 | [-.454] | [-.090] |  | -.30 | .001 | [-.471] | [-.122] |  | -.13 | .234 | [-.333] | [.082] |  |
| Conscientiousness x neuroticism | | | | | | | | | | | | | | | |  |
| E |  | .08 | .053 | [.001] | [.165] |  | -.01 | .850 | [-.089] | [.073] |  | .16 | .001 | [.067] | [.257] |  |
| O |  | .03 | .435 | [-.048] | [.111] |  | .02 | .689 | [-.062] | [.094] |  | .04 | .375 | [-.050] | [.132] |  |
| A |  | -.01 | .736 | [-.100] | [.071] |  | .01 | .967 | [-.081] | [.085] |  | -.03 | .569 | [-.127] | [.069] |  |
| C |  | .53 | .001 | [.368] | [.683] |  | .44 | .001 | [.289] | [.596] |  | .42 | .001 | [.238] | [.597] |  |
| C x N (m) |  | -.39 | .001 | [-.623] | [-.161] |  | -.21 | .074 | [-.430] | [.020] |  | -.47 | .001 | [-.734] | [-.208] |  |
| C x N (h) |  | -.34 | .001 | [-.537] | [-.134] |  | .18 | .077 | [-.373] | [.020] |  | -.40 | .001 | [-.632] | [-.173] |  |

Note. *N* = 480, CI = confidence interval. Low neuroticism was baseline value for all models. E = extraversion; O = openness to experience; A = agreeableness; C = conscientiousness; N = neuroticism; (m) = medium; (h) = high

**Table** **C. main and moderation effects for openness x neuroticism and agreeableness x neuroticism on general, inhibitory, and initiatory self-control**

| Predictor |  | General self-control | | | |  | Inhibitory self-control | | | |  | Initiatory self-control | | | |  |
| --- | --- | --- | --- | --- | --- | --- | --- | --- | --- | --- | --- | --- | --- | --- | --- | --- |
|  |  | *β* | *p* | 95% CI | |  | *β* | *P* | 95% CI | |  | *β* | *P* | 95% CI |  |  |
| Openness x neuroticism | | | | | | | | | | | | | | | |  |
| E |  | .07 | .084 | [-.010] | [.158] |  | -.01 | .761 | [-.094] | [.069] |  | .15 | .002 | [.057] | [.249] |  |
| O |  | .12 | .065 | [-.007] | [.245] |  | .08 | .225 | [-.047] | [.197] |  | .12 | .093 | [-.021] | [.268] |  |
| A |  | -.01 | .819 | [-.097] | [.076] |  | .01 | .889 | [-.078] | [.089] |  | -.03 | .617 | [-.124] | [.074] |  |
| C |  | .28 | .001 | [.185] | [.366] |  | .31 | .001 | [.224] | [.399] |  | .12 | .028 | [.013] | [.220] |  |
| O x N (m) |  | -.13 | .172 | [-.321] | [.057] |  | -.15 | .216 | [-.297] | [-.067] |  | -.09 | .406 | [-.307] | [.125] |  |
| O x N (h) |  | -.12 | .159 | [-.294] | [.048] |  | -.07 | .410 | [-.235] | [.096] |  | -.13 | .177 | [-.331] | [.061] |  |
| Agreeableness x neuroticism | | | | | | | | | | | | | | | |  |
| E |  | .07 | .085 | [-.010] | [.158] |  | -.01 | .769 | [-.092] | [.067] |  | .15 | .002 | [.056] | [.248] |  |
| O |  | .04 | .305 | [-.038] | [.123] |  | .02 | .563 | [-.054] | [.100] |  | .05 | .272 | [-.041] | [.144] |  |
| A |  | .07 | .337 | [-.072] | [.209] |  | .10 | .168 | [-.040] | [.231] |  | .01 | .904 | [-.151] | [.171] |  |
| C |  | .28 | .001 | [.191] | [.372] |  | .32 | .001 | [.229] | [.403] |  | .12 | .020 | [.020] | [.226] |  |
| A x N (m) |  | -.16 | .110 | [-.359] | [.036] |  | -.16 | .105 | [-.348] | [.331] |  | -.10 | .386 | [-.327] | [.126] |  |
| A x N (h) |  | -.10 | .303 | [-.293] | [.091] |  | -.13 | .166 | [-.316] | [.054] |  | -.03 | .817 | [-.246] | [.194] |  |

Note. *N* = 480, CI = confidence interval. Low Neuroticism was baseline value for all models. E = extraversion; O = openness to experience; A = agreeableness; C = conscientiousness; N = neuroticism; (m) = medium; (h) = high.

Finally, we examined to what extent neuroticism moderated the relationship between conscientiousness and general self-control. As in the main article, Table C show that neuroticism moderated this relationship (*p* < .001). The total ANOVA analysis favored the interaction model, *F*(2, 469) = 7.19, *p* < .001. Furthermore, the regression analysis for the interaction model yielded an *R^2^* of .20, *F*(10, 469) = 12.70, *p* < .001.

**Inhibitory and initiatory self-control – the moderating effect of neuroticism**

In this section we report results regarding to what extent neuroticism moderated the relationship between the four personality traits, extraversion, openness to experience, agreeableness, and conscientiousness, on the one hand, and inhibitory and initiatory self-control on the other.

As in the main article and shown in Table B, neuroticism moderated the relationship between extraversion and *inhibitory* self-control (*p* < .001). A one-way ANOVA compared models with and without interactions terms, and favored the interaction model, *F*(2, 469) = 7.03, *p* < .001. The regression analysis with interaction terms yielded an *R*^2^ of .21, *F*(10, 469) = 13.99, *p* < .001.

As in the main article, we found no moderator effect of neuroticism on the relationship between extraversion and *initiatory* self-control (Table B), indicating that different levels of neuroticism do not significantly influence the relationship between extraversion and initiatory self-control.

Table C show that neuroticism did neither moderate the relationships between openness to experience on the one hand and inhibitory or initiatory self-control on the other, nor the relationship between agreeableness and the two other types of self-control.

As in the main article, Table B show that there was no moderation effect of neuroticism on the relationship between conscientiousness and *inhibitory* self-control, indicating that different levels of neuroticism do not significantly influence the relationship between conscientiousness and inhibition.

As in the main article, Table B show that neuroticism moderated the relationship conscientiousness and *initiatory* self-control (*p* < .001). The total ANOVA favored the interaction model, *F*(2, 469) = 7.95, *p* < .001. Moreover, the regression analysis for the interaction model yielded an *R*^2^ of .09, *F*(10, 469) = 5.75, *p* < .001.

**Conclusion**

The robustness check, which included all four remaining personality traits of the Five-Factor Model in the analysis of how neuroticism moderates the relationship between personality traits and self-control, yielded results consistent with those in the main article. This supports the decision to exclude openness to experience and agreeableness in the main analyses.
